# Vacuum and Low-Temperature Characteristics of Silicon Oxynitride-Based Bipolar RRAM

**DOI:** 10.3390/mi13040604

**Published:** 2022-04-12

**Authors:** Nayan C. Das, Minjae Kim, Sung-Min Hong, Jae-Hyung Jang

**Affiliations:** 1 Gwangju Institute of Science and Technology, School of Electrical Engineering and Computer Science, Gwangju 61005, Korea; nayan@gm.gist.ac.kr (N.C.D.); min7kim9@gm.gist.ac.kr (M.K.); smhong@gist.ac.kr (S.-M.H.); 2 Korea Institute of Energy Technology, School of Energy Technology, Naju 58330, Korea

**Keywords:** bipolar, cryogenic temperature, operating ambiances, RRAM

## Abstract

This study investigates the switching characteristics of the silicon oxynitride (SiO_x_N_y_)-based bipolar resistive random-access memory (RRAM) devices at different operating ambiances at temperatures ranging from 300 K to 77 K. The operating ambiances (open air or vacuum) and temperature affect the device’s performance. The electroforming-free multilevel bipolar Au/Ni/SiO_x_N_y_/p^+^-Si RRAM device (in open-air) becomes bilevel in a vacuum with an on/off ratio >10^4^ and promising data retention properties. The device becomes more resistive with cryogenic temperatures. The experimental results indicate that the presence and absence of moisture (hydrogen and hydroxyl groups) in open air and vacuum, respectively, alter the elemental composition of the amorphous SiO_x_N_y_ active layer and Ni/SiO_x_N_y_ interface region. Consequently, this affects the overall device performance. Filament-type resistive switching and trap-controlled space charge limited conduction (SCLC) mechanisms in the bulk SiO_x_N_y_ layer are confirmed.

## 1. Introduction

Low-temperature electronics technology has the potential to improve device and circuit performances. When semiconductor devices are operated at a cryogenic temperature (approximately 123 K and below), the system performance can be improved [1]. Moreover, recent research interest in quantum computing, space electronics, and superconducting circuits has also led to developments in cryogenic data storage technology [2,3]. However, one of the significant challenges to implementing cryogenic electronics is finding a suitable and compatible cryogenic memory that can operate at cryogenic temperatures [2,3,4,5].

Resistance-based (non-superconducting) memories offer better scalability, faster speed, and lower power consumption than charge-based memories among different cryogenic memory technologies. Moreover, resistance-based memories provide nonvolatility. Among the resistance-based memories, resistive random access memory (RRAM) devices are strong candidates for a cryogenic memory due to their low-power operation, excellent scalability, high reliability, and simple manufacturing processes [2,3,4,5,6,7].

Although extensive studies have been conducted on RRAM devices, most studies were completed in open air at room temperature. Few studies investigated the effects of air, oxygen, and nitrogen partial pressure on RRAM device performance by varying operating ambiance [6,8,9]. Studies also showed that the electroforming process is not possible in a vacuum for SiO_2_-, Ta_2_O_5_-, and HfO_2_-based devices [10,11,12,13,14]. Only two binary oxide material (HfO_x_ and Cu-doped silica)-based RRAM devices have been studied at cryogenic temperatures to date [4,5,15,16,17,18,19]. All other studies (except for Shang et al. [18]) required an electroforming process to activate the resistive switching properties in open air at room temperature before measuring device performance cryogenic temperatures [4,5,15,16,17,18,19]. Even though cryogenic temperature memory devices ideally require temperature-independent properties, this has not yet been explored [20].

In order to overcome the limitations mentioned above, a reliable RRAM device, which works in a vacuum at cryogenic temperatures, is needed. Thus, it is essential to study in detail the interaction of the RRAM device with the ambience (open air and vacuum), when varying the temperature from room temperature to cryogenic temperatures. After developing a multilevel bipolar electroforming-free resistive switching memory (Au/Ni/SiO_x_N_y_/p^+^-Si device) in open air at room temperature [21], the next obvious step is to explore the feasibility of the device in a vacuum and at cryogenic temperatures.

This work reports that Au/Ni/SiO_x_N_y_/p^+^-Si RRAM devices are operable at cryogenic temperatures without pretreatment. They exhibit favorable resistive switching properties in the ambience at room temperature and at temperatures ranging from 77 K to 300 K. The change in operating ambience influences the performance of the devices. Depending on the operating environment, the availability of the weakly bonded hydroxyl groups influences the surface chemistry of the amorphous SiOxNy active layer and the Ni/SiO_x_N_y_ interface, which subsequently changes the elemental composition of the Ni/SiO_x_N_y_ interface region and affects the device performance.

## 2. Materials and Methods

The 10 nm thick SiO_x_N_y_ thin films were deposited by plasma-enhanced chemical vapor deposition (PECVD) on a p^+^-Si substrate (boron-doped, 3.0 mΩ∙cm) at 300 °C. The gas mixture, N_2_O/SiH_4_/NH_3_/N_2_ (60/400/20/600 SCCM), was supplied under a working pressure of 650 mTorr and an RF power of 15 W (30.6 W/mm^2^). A circular-shaped Au/Ni (150/40 nm) top electrode with a radius of 50 μm was deposited by e-beam evaporation through a shadow mask to complete the device fabrication. The heavily doped p^+^-Si substrate served as the bottom electrode. For X-ray photoelectron spectroscopy (XPS; NEXSA, Fisher Scientific, 168 Third Avenue. Waltham, MA, USA) and Fourier-transform infrared (FTIR) absorbance spectroscopy measurement, 50 nm and 1 μm thick SiO_x_N_y_ films were grown separately. The IR spectra of SiO_x_N_y_ films under vacuum and ambient conditions were measured by a Vertex 80v IR spectrometer equipped with an infrared reflection absorbance (IRRAS) accessory (Bruker A513, incident angle 45°, Bruker Optics, Ettlingen, Germany) and MCT detector. The pressure in the vacuum was about 1.27 hPa (9.5 × 10^−1^ torr).

The electrical characteristics of the memory devices were measured using a semiconductor parameter analyzer (HP-4155A; Hewlett-Packard Company, 3000 Hanover Street, Palo Alto, CA, USA). The electrical characteristics of RRAM devices in a vacuum environment and at cryogenic temperatures were measured using an MS-TECH Vacuum Chamber Probe Station (<10^−5^ torr). A voltage was applied directly to the top electrode, while the bottom electrode was grounded.

Three different batches of samples for each sample type were analyzed to confirm the reproducibility. A batch consisted of more than 20 devices, and more than 50 devices were measured in each condition (in a vacuum at 300 K to 77 K) to confirm the observations and conclusions. Due to the few process variables involved in the device fabrications and considering that each process condition was well controlled, the performance of different devices in the same batch and different batches was almost the same. Additionally, all devices showed similar cycle-to-cycle variations. The range of device-to-device variation was smaller than that of cycle-to-cycle variation. 

## 3. Results and Discussion

### 3.1. Properties of SiO_x_N_y_ Film

**Figure 1:**
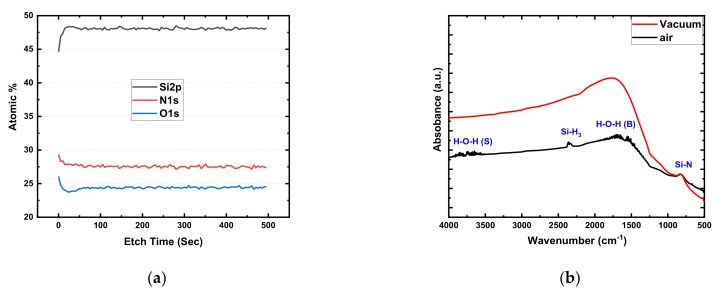
Compositional analysis of SiO_x_N_y_ thin film: (**a**) XPS depth profile with atomic percentages; (**b**) FTIR absorbance spectra measured in open-air and vacuum environments.

Compositional analysis of the PECVD grown amorphous SiO_x_N_y_ film is shown in Figure 1. The XPS depth profile analysis shows that the Si/N/O atomic percentage (45.52%, 28.91%, and 25.58%) and ratio (1:0.64:0.56) of the as-deposited SiO_x_N_y_ film were constant throughout the film, indicating a sufficient number of oxygen vacancies and nitride-related traps in the layer (Figure 1a).

FTIR absorbance spectra for the SiO_x_N_y_ film measured in open-air and vacuum environments are shown in Figure 1b. The stable characteristic absorbance peak of the Si–N bond was found at 831 cm^−1^ for both measurement conditions. However, many weak absorption peaks were observed in the ranges 3800–3500 cm^−1^ and 1700–1450 cm^−1^, associated with the weak binding between Si^2+^ sites and different vibrational modes (stretching and bending) of hydroxyl groups in open-air measurement conditions [22,23,24,25,26].

In SiO_x_N_y_, the Si–OH bond vibrations typically peaked at around 3500 cm^−1^, and the band of N–H bonds peaked at about 3320 cm^−1^. The absorption band in the range of 2300–2390 cm^−1^ was due to the Si–H stretching vibrations [26]. Si–H_3_ vibration resulted in an absorption band around 2255 cm^−1^, and this band could shift further to a higher wavenumber if more nitrogen atoms are introduced into the thin film [26]. These hydroxyl groups indicated the presence of H_2_O on the surface of the amorphous SiO_x_N_y_ that was absorbed from the ambience during the fabrication process. In a vacuum environment, all weakly bonded hydrogen and hydroxyl groups are easily removed from the surface of the amorphous SiO_x_N_y_ thin film [27,28,29].

### 3.2. Electrical Characteristics of Au/Ni/SiO_x_N_y_/p^+^-Si Device

The viability of the Au/Ni/SiO_x_N_y_/p^+^-Si memory device at cryogenic temperature was investigated in two steps. At first, the device performances were measured in a vacuum chamber and at room temperature. After that, the device performance was measured in a vacuum chamber at 77 K.

#### 3.2.1. Device Performance in a Vacuum at Room Temperature

**Figure 2:**
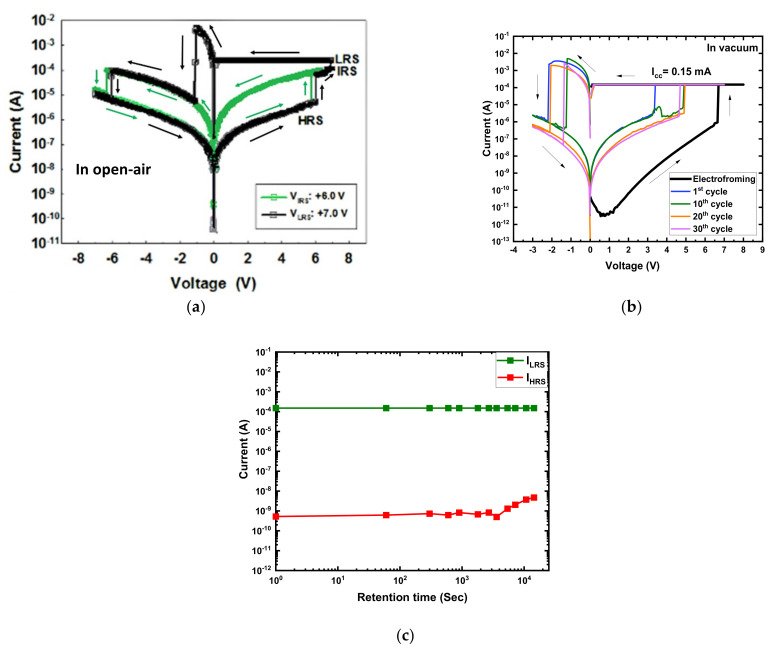
Typical I–V characteristics of Au/Ni/SiO_x_N_y_/p^+^-Si at room temperature (**a**) in open air [21], and (**b**) in a vacuum; (**c**) data retention characteristics of the device in a vacuum at 300 K.

In the open-air environment, the current–voltage (I–V) measurement of the Au/Ni/SiOxNy/p^+^-Si devices with a 50 μm radius was conducted by applying double-sweep DC voltage in the sequence of 0 V → +7 V → 0 V → −7 V → 0 V with a 0.25 mA compliance current (I_cc_) and a 50 mV step. The electroforming-free bipolar multilevel switching operation of the 10 nm thick SiO_x_N_y_-based device (black) is shown in Figure 2a. The stability of the intermediate resistance state (IRS) was confirmed by cycling 0 V → +6.5 V → 0 V → −7 V → 0 V, which is shown in green (Figure 2a). A detailed study of the multilevel electroforming-free bipolar resistive switching behavior of the devices was reported separately [21].

**Figure 3:**
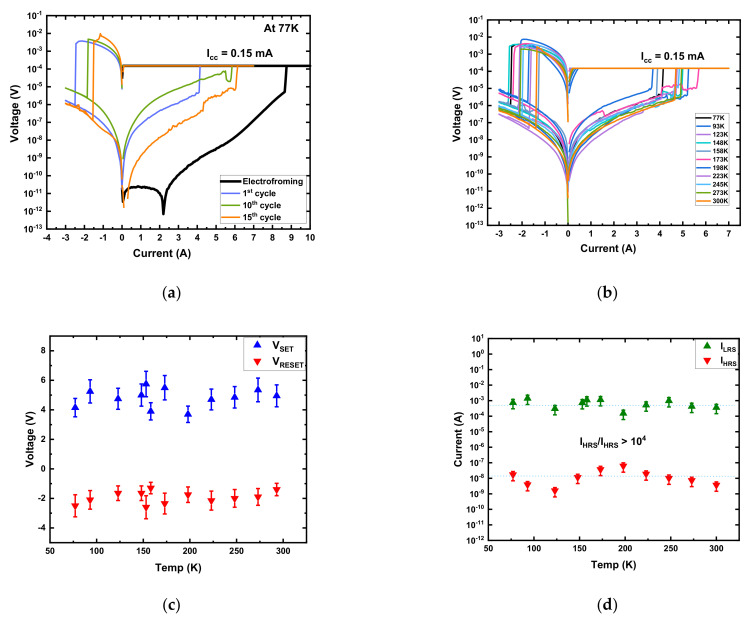
Au/Ni/SiO_x_N_y_/p^+^-Si memory device performance in a vacuum. (**a**) Typical I–V curves at 77 K; (**b**) typical I–V curves with temperature variation from 77 K to 300 K; (**c**) V_SET_ and V_RESET_ vs. temperature; (**d**) I_LRS_ and I_HRS_ vs. temperature.

I–V measurement of the devices was carried out when the chamber was in a vacuum (<10^−3^ torr) at 300 K to investigate the effects of the operating environment on the device performance. Figure 2b shows the I–V characteristics of the device in a vacuum at 300 K. In the vacuum environment, basic operating parameters such as the 0.15 mA I_cc_ and 50 mV step were kept constant. The first double-sweep DC voltage in the 0 V → +8 V → 0 V → −3 V → 0 V was applied for the electroforming process. When the positive bias voltage was applied, the current increased sharply at the electroforming voltage (V_FORM_) around +7 V, and the device reached the low resistance state (LRS). The device returned to the new high resistance state (HRS) when a negative voltage was applied. After electroforming, the sequence was changed to 0 V → +7 V → 0 V → −3 V → 0 V (Figure 2b). The SET process occurred in the range of +3.2 to +5.0 V (V_SET_), and the RESET process occurred at the RESET voltage (V_RESET_) in the range of −1.30 to −2.15 V. The LRS current (I_LRS_) and HRS current (I_HRS_) values of the device were read at −0.50 V (V_READ_). The data retention characteristics of the device in a vacuum at 300 K are shown in Figure 3c. The device exhibited promising data retention over 10^4^ s with an on/off ratio higher than 10^4^.

The multilevel Au/Ni/SiO_x_N_y_/p^+^-Si RRAM device became bilevel with an on/off ratio >10^4^ when the operating ambience was changed from open air to vacuum. The device also exhibited three-order-higher initial resistance (~GΩ) in a vacuum at room temperature than the device (~MΩ) measured in the open-air environment. The pristine device requires an electroforming process to activate resistive switching properties in a vacuum where the device operates electroforming-free in open air at 300 K. The electroforming-free characteristic of the device in open air is caused by the combined effects of sufficient internal defects (oxygen vacancies and nitride-related traps) and the presence of external defects (O–H, and –H groups) on the surface of the amorphous SiO_x_N_y_ active layer. The O–H groups provide additional charges and facilitate the formation of anion vacancies at the interface of the Ni/SiO_x_N_y_ [11,14,30,31,32]. In a vacuum, weakly bonded hydrogen and hydroxyl groups are removed from the surface of the amorphous SiO_x_N_y_ thin film, which makes the active layer more resistive; the overall initial resistance of the device increases by a factor of three, and the IRS disappears [10,11,12,13,14].

The effects of the device size (area) and the active layer thickness on the device performance were explained in detail in a previous study [21]. The I_LRS_ independence of the area implies that the current conduction was dominated by filamentary conduction [21,33]. The V_SET_ and V_RESET_ active layer thickness dependencies show that filament-type resistive switching was bulk-dependent, indicating that a conductive filament (CF) was formed from the bottom electrode to the top electrode through the bulk SiO_x_N_y_ layer [21,33].

After electroforming, the CF dissolved partially at its weak point during the RESET process, and the device reached a new HRS, which was less resistive than the initial HRS. Further SET/RESET processes happened through reconstruction and partial breakdown of the CF, requiring a smaller magnitude of voltages (|V_SET_| and |V_RESET_|) than the |V_FORM_| [34]. 

#### 3.2.2. Device Performance in a Vacuum at Cryogenic Temperature

The resistive switching performance of the Au/Ni/SiO_x_N_y_/p^+^-Si memory devices in a vacuum (<10^−5^ torr) at 77 K is shown in Figure 3a. For the first sweep, DC voltage was applied in the sequence of 0 V → +10 V → 0 V → −3 V → 0 V. The pristine device was in the HRS with a resistance of 100 GΩ at a low temperature, which is much higher than the resistance (~10 GΩ) in vacuum at room temperature. V_FORM_ (≈+8.5 V) was also higher than in vacuum at 300 K (≈+7 V). The cryogenic temperature made the device more resistive due to the semiconducting behavior, indicating the possible involvement of the thermionic conduction process. The formation and dissolution of a CF include charge transfer, ion motion, and nucleation, which are affected by the change in temperature [4]. With the reduction in temperature to 77 K, the thermal energies of oxygen vacancies and ions are also reduced. Consequently, the device needs a higher V_FORM_ to form CFs [5,17]. As a result, increases in the initial resistance and V_FORM_ were observed at low temperatures.

During the electroforming process in vacuum, both at 300 K and at 77 K, there was an abnormal dip in the current at a positive bias side, as shown in the black in Figure 2b and Figure 3a. The abnormal dips were attributed to the negative differential resistance (NDR) effect. In general, a higher bias voltage corresponds to a larger current. However, the phenomenon when the current decreases with the increase in bias voltage is called NDR, which has been observed during the electroforming or set process [35,36,37,38,39]. The NDR phenomena are mainly observed in RRAM devices due to the trap/de-trap of electronic carriers between deeply localized states induced by implanted metal nanoparticles [36,37] or the accumulation of defects caused by junction reinstallment [35]. They can also be observed due to the recombination between oxygen vacancies and the thermally released oxygen ions from the oxide interface layer [38,39]. In the Au/Ni/SiO_x_N_y_/p^+^-Si device, the NDR phenomena were mainly observed during the electroforming process due to the trapping of electronic carriers in the deeply localized states induced by oxygen vacancies and nitride-related traps in the fresh device.

After electroforming, the cycle sequence was 0 V → +7 V → 0 V → −3 V → 0 V. V_SET_, V_RESET_, I_LRS_, and I_HRS_ followed a similar pattern to that in vacuum at room temperature (Figure 3a). The device performed well with a minimum on/off ratio >10^4^ at 77 K.

At 77 K, the variability in the I–V curves of the device performance resulted from different CF configurations of oxygen vacancies and nitride-related traps between the Ni and p^+^-Si with varying heights of the barrier due to the different bias cycles [5]. At 77 K, the LRS also showed more uniformity than HRS.

The I–V characteristics were measured from 77 K to 300 K after electroforming at 77 K to investigate the effect of the temperature variation on the device’s performance (Figure 3b). Voltages (V_SET_ and V_RESET_) and currents (I_LRS_ and I_HRS_) as a function of temperatures are presented in Figure 3c,d, respectively.

As demonstrated in Figure 3c,d, after electroforming, the random variation in V_SET_, V_RESET_, I_LRS_, and I_HRS_ implies that the resistive switching characteristics were less affected by temperature variation than cycle-to-cycle variation. The variation in V_RESET_ and V_SET_ indicates that the CFs were partially ruptured after RESET [4,40]. The partially ruptured CF in the RESET process was attributed to the combined effects of the oxygen vacancy recombination with nitride-related traps by electric fields (primarily) and localized Joule heating (assisting) [19,33,41,42]. Joule heating can subdue the operating ambient temperature effect as the local temperature due to Joule heating can surpass the ambient temperature by a significant amount [20,42,43]. As a result, after electroforming, V_SET_, V_RESET_, I_LRS_, and I_HRS_ of the device were less affected by the temperature variation, providing these potential devices with stability in the cryogenic temperature range, appropriate for applications using RRAM technology [44].

#### 3.2.3. Conduction and Switching Mechanism in Vacuum at 77 K

**Figure 4:**
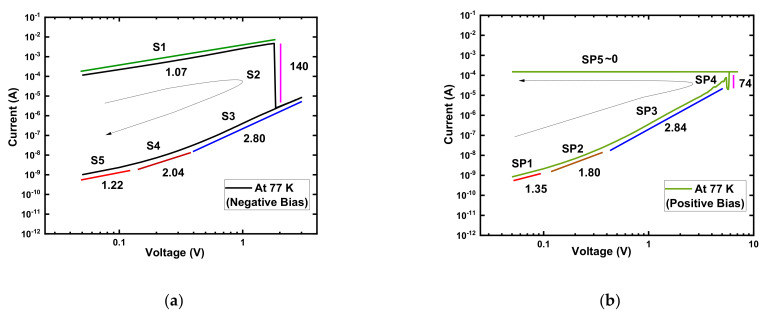
Log (I)–log (V) characteristics of Au/Ni/SiO_x_N_y_/p^+^-Si memory devices with I_cc_ = 0.15 mA at 77 K. (**a**) Negative bias voltage region; (**b**) positive bias voltage region with slopes of different parts.

A typical I–V curve from Figure 3a was replotted as log(I)−log(V) with curve fittings to explore the current conduction process of the Au/Ni/SiO_x_N_y_/p^+^-Si device at 77 K after electroforming in Figure 4. The HRS and LRS of the negative voltage regions were divided into S1, S2, S3, S4, and S5 (Figure 4a). Similarly, the positive voltage regions were divided into SP1, SP2, SP3, SP4, and SP5 (Figure 4b).

In the negative voltage region, the slope of the LRS (S1: 1.07) indicated ohmic conduction (I ∝V). At the RESET voltage region (S2: 140), the conduction mechanism followed Child’s law ((I∝V*^n^* where *n* = 4–150). At higher voltages, the slopes of the HRS (S3: 2.8, S4: 2.04) followed Child’s law ((I∝V*^n^* where *n* = 1.5–3). The positive voltage region also showed a similar pattern even though the I_cc_ limit controlled the LRS current. In the negative and positive low-voltage regions, the slopes (S5: 1.22, SP1: 1.35) were slightly higher than 1, which can be attributed to the incomplete rupture and formation of the CF during the RESET and SET processes after electroforming at low temperatures.

The above analysis and an earlier study [21] showed that the conduction mechanism for the device is always the trap-controlled SCLC regardless of operating environment and temperature [21,45,46,47,48].

**Figure 5:**
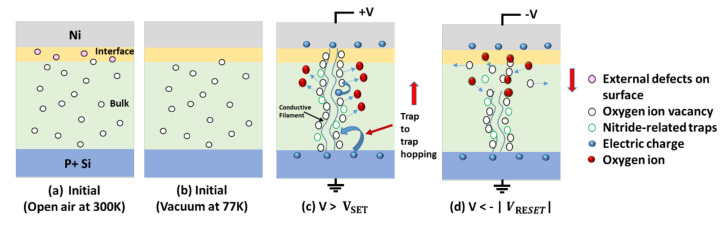
Schematics of proposed switching mechanism of Au/Ni/SiO_x_N_y_/p^+^-Si memory device in a vacuum at 77 K.

According to the current conduction mechanism analysis, the resistive switching mechanism with the schematics of the Au/Ni/SiO_x_N_y_/p^+^-Si memory device in cryogenic temperature is proposed below (Figure 5).

In an open-air environment, the as-prepared device contained a sufficient number of internal defects and external defects. The internal defects were a combination of silicon dangling bonds, oxygen vacancies, and nitride-related traps in the bulk of the initial SiOxNy layer [49] (Figure 5a). These silicon dangling bonds and oxygen vacancies play major roles in resistive switching [21,25,50]. The external defects were the weakly bonded O–H groups on the surface of SiO_x_N_y_, facilitating the creation of anion vacancies at the interface by disassociating into differently charged species (O^2−^ and H^+^). Thus, the presence of the ionic charge carriers at the interface region was altered from the bulk SiO_x_N_y_ and increased the interface region’s conductivity [11,14,30,31]. All defects (internal and external) in the devices were considered as traps (Figure 5a). The external defects (hydrogen and hydroxyl groups) were removed from the Ni/SiO_x_N_y_ interface in vacuum. At 77 K, the pristine device became more resistive due to the reduction in thermal energies of ions and oxygen vacancies [5,17] (Figure 5b). The switching mechanism was fundamentally related to the oxygen and nitrogen content in the SiO_x_N_y_ layer. When the positive voltage increased beyond the V_FORM_, CFs of oxygen vacancies were formed between the Ni and p^+^-Si through the bulk SiO_x_N_y_ layer in the electroforming process (not explicitly shown in Figure 5), and the device resistance state changed from HRS to LRS. The Ni layer acted as a charge reservoir, and oxygen ions traveled by hopping through the nitride-related traps of the SiO_x_N_y_ layer under the applied positive voltage. After the initial formation of a CF, electronic carriers hopped through the oxygen vacancy and nitride-related trap-based CFs at V_SET_ (Figure 5c). When the negative bias voltage approached V_RESET_, the oxygen ions traveled back from the Ni layer and recombined with the oxygen vacancies. This capture process broke the oxygen vacancy-based CFs, leading to resistive switching from the LRS to HRS (Figure 5d) [21].

## 4. Conclusions

Fully functional Au/Ni/SiO_x_N_y_/p^+^-Si memory devices were successfully fabricated, which could operate in open-air and vacuum environments at temperatures ranging from 300 K to 77 K. In an open-air laboratory environment, the device showed electroforming-free multilevel bipolar resistive switching. However, the multilevel RRAM device became bilevel in a vacuum with an on/off ratio >10^4^ at room temperature. The device showed promising stability with data retention higher than 10^4^ s. At 77 K, the device became more resistive than at room temperature, but a high on/off ratio (>10^4^) was maintained. The presence (in the atmospheric environment) and absence (in vacuum) of moisture (hydrogen and hydroxyl groups)-related defects at the amorphous SiO_x_N_y_ active layer and the Ni/SiO_x_N_y_ interface were determined by the operating environment. These hydrogen and hydroxyl groups subsequently changed the Ni/SiO_x_N_y_ interface region’s elemental compositions and affected the device performance.
